# Supplementary material for: Functional annotation of proteins for signaling network inference in non-model species
Source: Nat Commun. 2023 Aug 3;14:4654. doi: 10.1038/s41467-023-40365-z (PMC10400656; doi:10.1038/s41467-023-40365-z)
Supplement: Supplementary file 1 — Supplementary Information [file 41467_2023_40365_MOESM1_ESM.pdf]

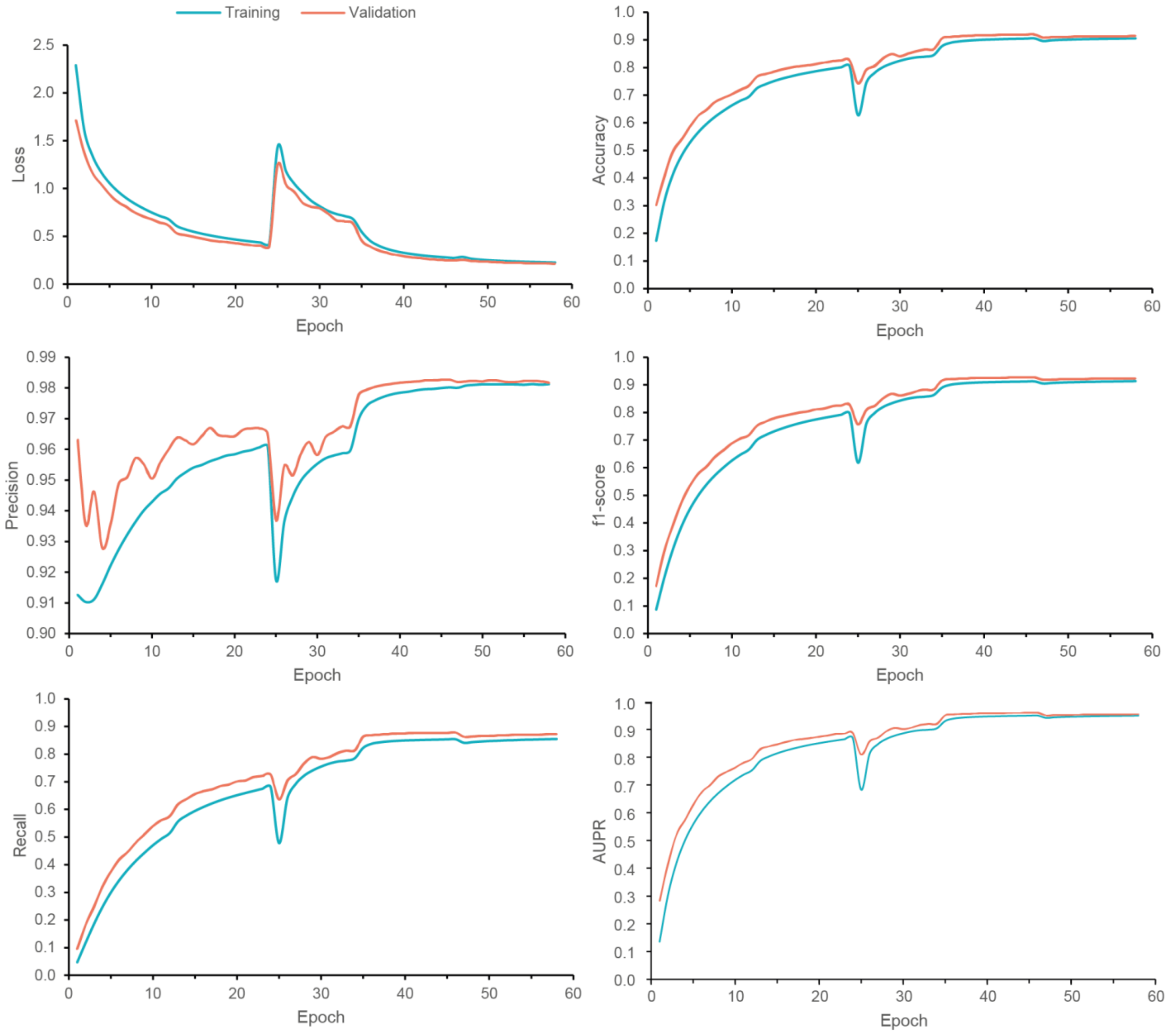

Supplementary Fig. S1 - Classification performance of PF-NET. The overall performance of PF-NET in terms of loss, accuracy, precision, f1-score, recall, and area under the precision curve (AUPR) during training and validation.

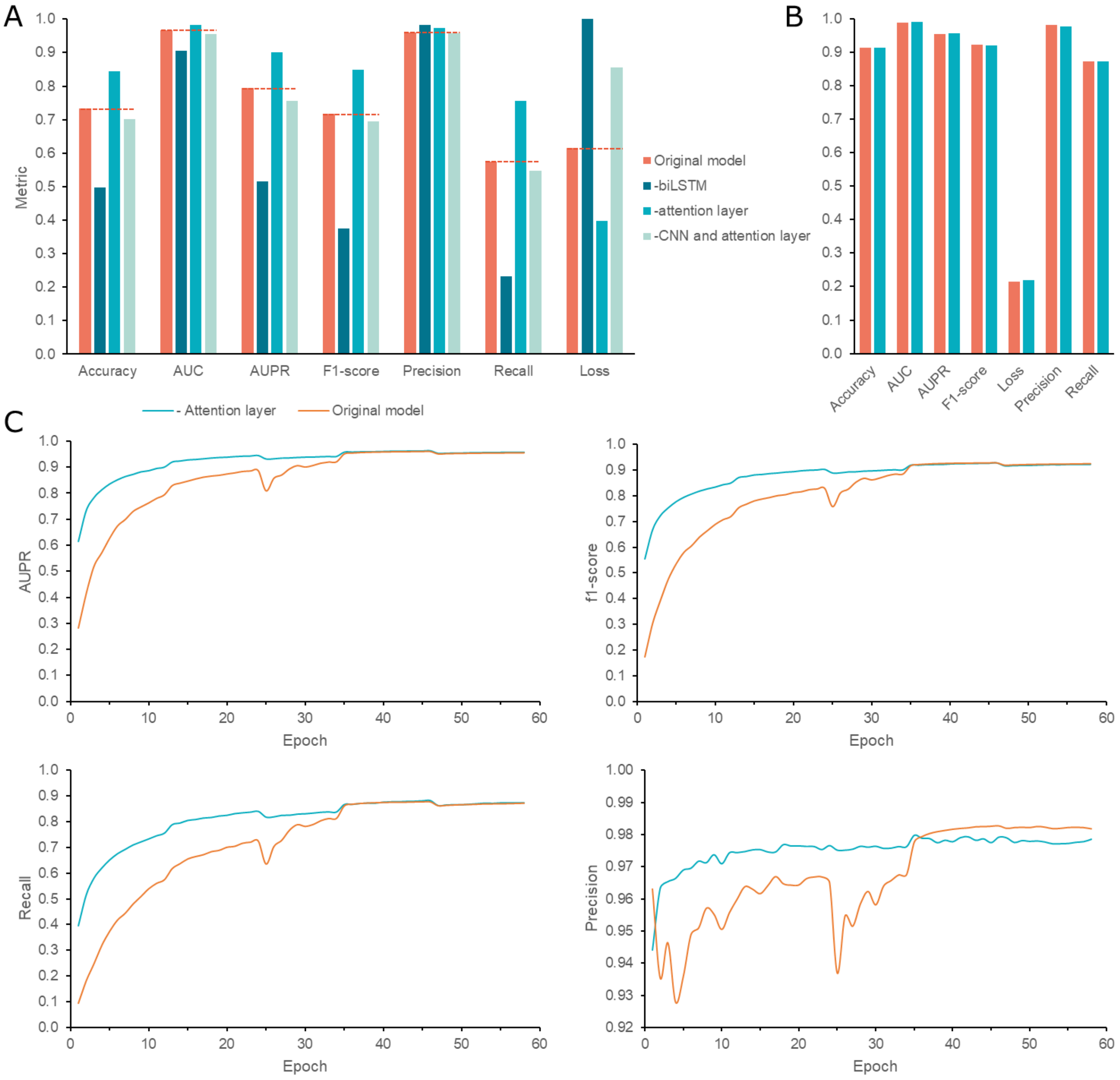

Supplementary Fig. S2 – Neural network performance upon an ablation study of each of its three layers. (A) The performance of PF-NET (original model) and three models where the biLSTM, the attention layer, and both the CNN and attention later are removed. The performance is given of the validation dataset after training with one batch combination of sequences (~ 3.6 million sequences) (B) The performance of PF-NET and the same model without attention layer after training on the full five batch combinations. (C) The overall performance of the validation dataset of the models in B in terms of area under the precision curve (AUPR) , f1-score, recall, and precision during training.

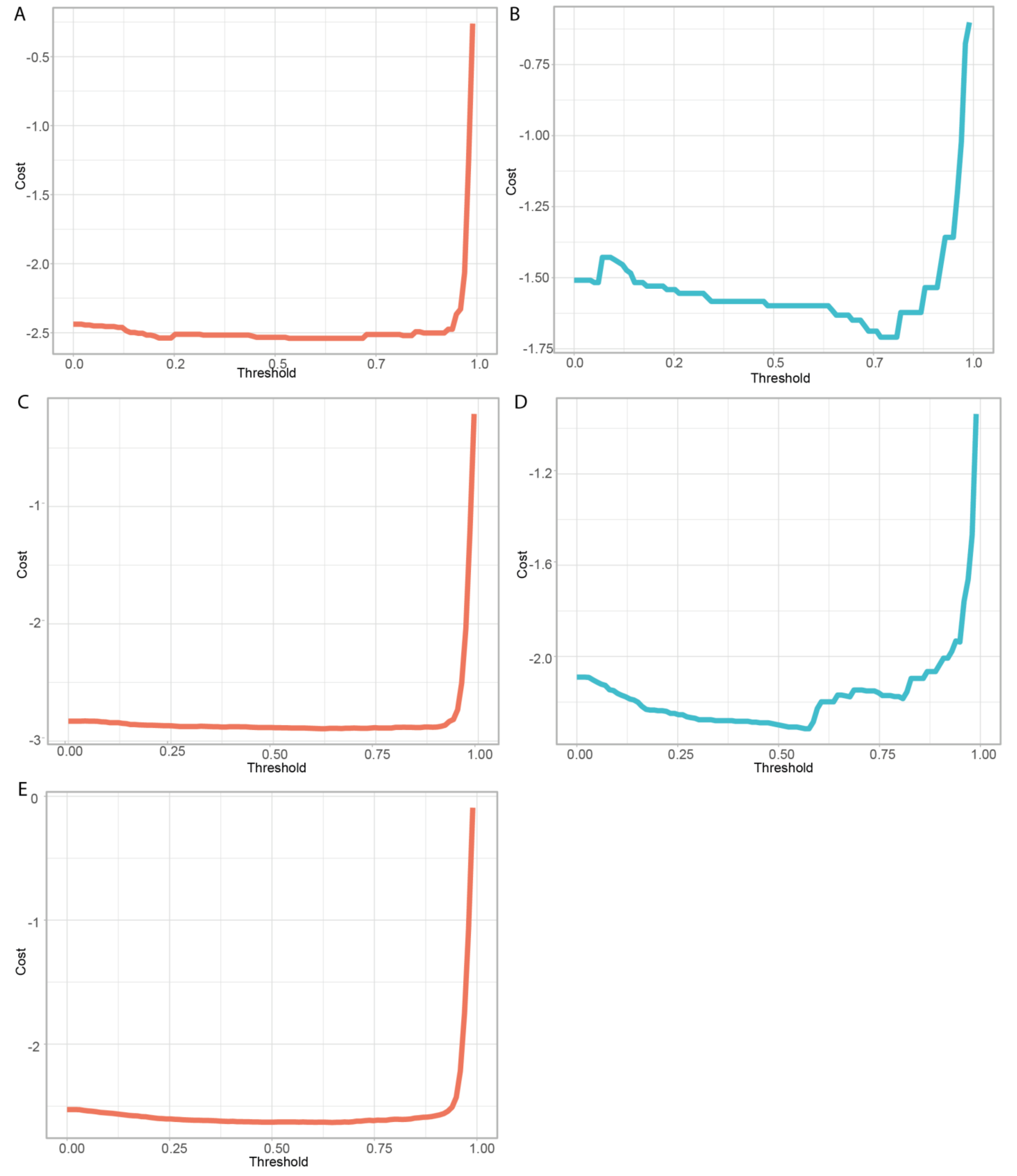

Supplementary Fig. S3 - Network performance on the yeast, Arabidopsis, and soybean kinases and phosphatases at different prediction probability thresholds. (A-E) The cost was calculated for 100 probability thresholds for the predicted *Saccharomyces cerevisiae* kinases (A) and phosphatases (B), *Arabidopsis thaliana* kinases (C) and phosphatases (D), and the *Glycine max* kinases (C).

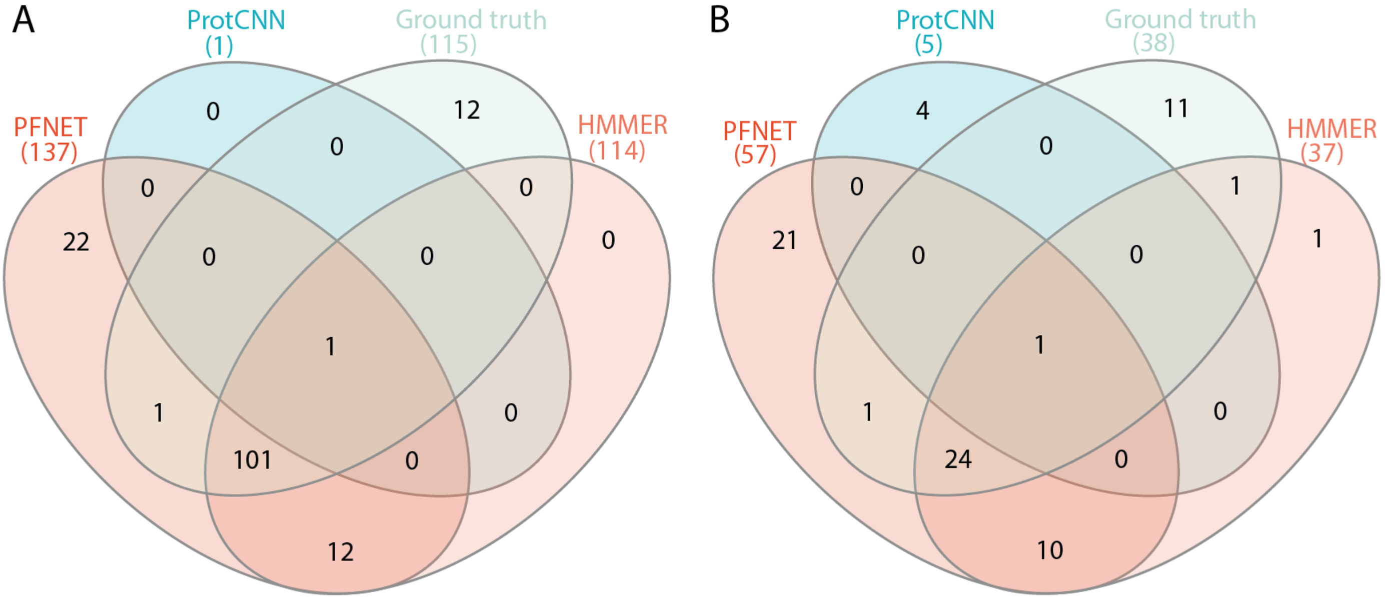

C

	Kinases			Phosphatases		
	PF-NET	HMMR	ProtCNN	PF-NET	HMMR	ProtCNN
Sensitivity	89%	89%	1%	66%	68%	3%
FDR	12%	11%	0%	24%	30%	11%

Supplementary Fig. S4 - Functional predictions of kinases and phosphatases in *Saccharomyces cerevisiae*. (A-B) Commonly identified kinases (A) and phosphatases (B) by PF-NET, HMMER search, ProtCNN, and the ground truth. (C) The overall performance of PF-NET and HMMER in terms of sensitivity and false discovery rate (FDR).

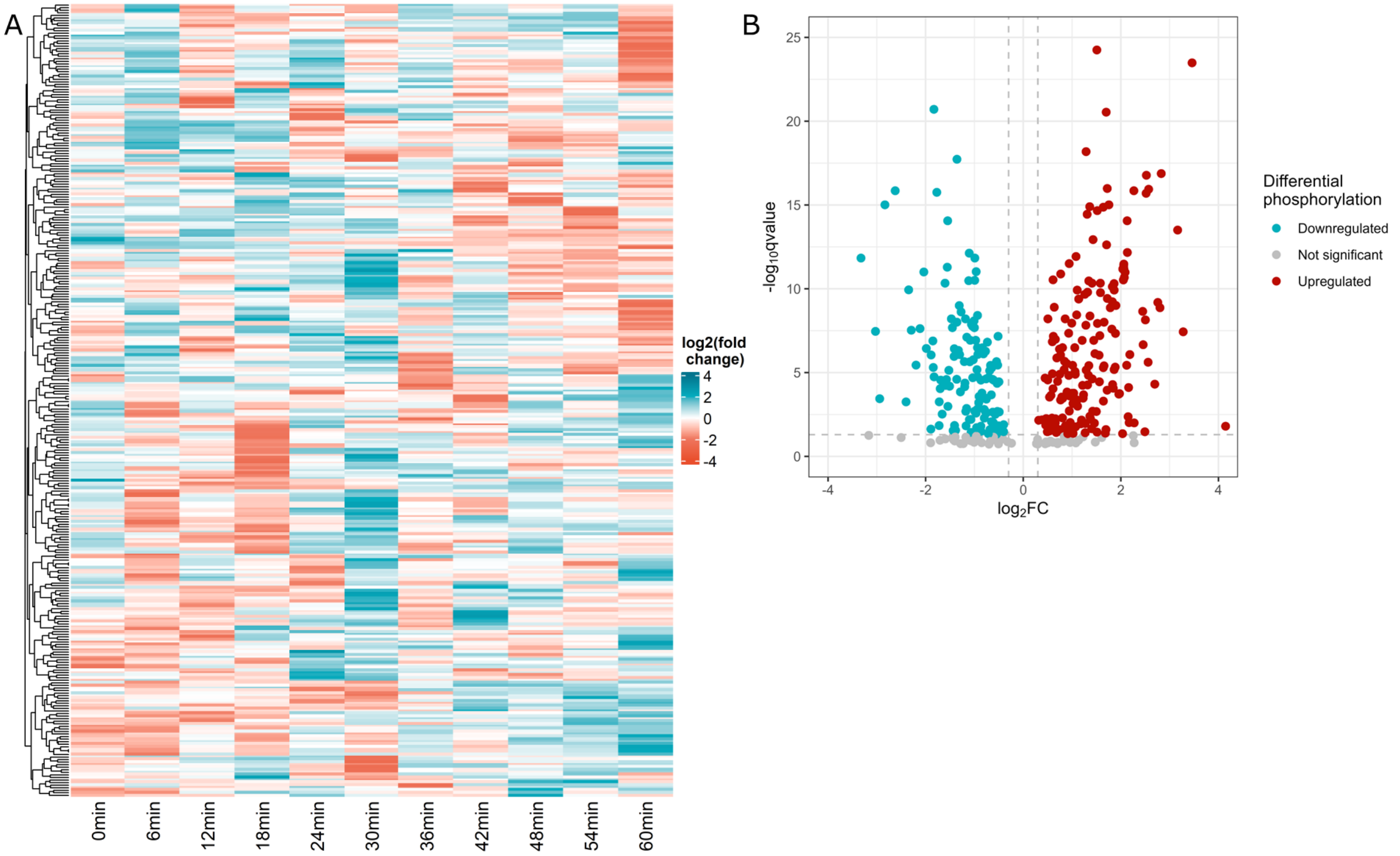

Supplementary Fig. S5 - Significant differentially phosphorylated phosphopeptides upon cold in soybean. (A) Heatmap of the $\log_2$ fold change upon cold across the entire time course. (B) A volcano plot that displays the maximum fold change of the time course in respect to the $-\log_{10}$ qvalue.

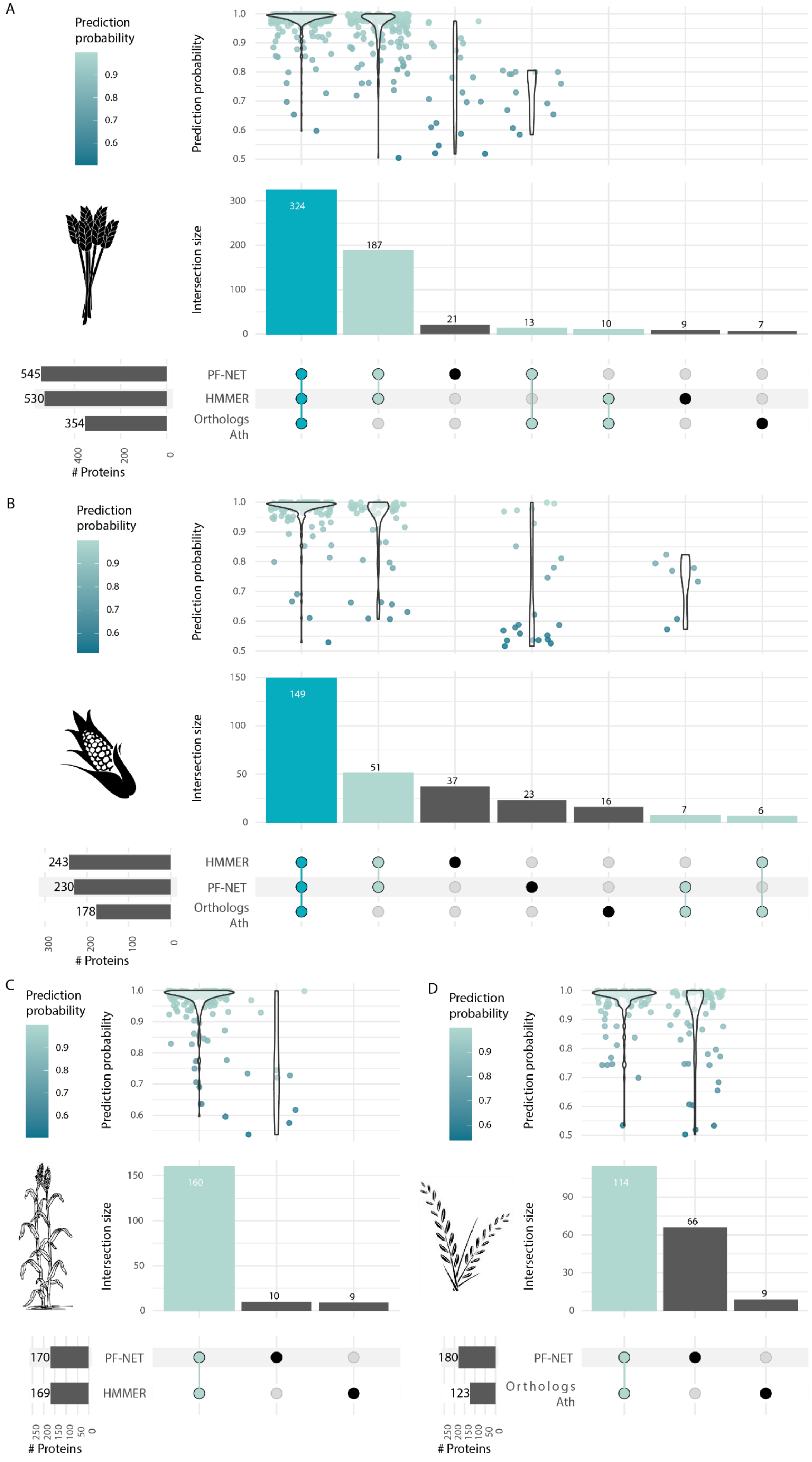

Supplementary Fig. S6 - Commonly identified phosphatases by PF-NET, HMMER search, and ortholog search in non-model species. (A-D) For *Triticum aestivum* (wheat) (A), *Zea mays* (maize) (B), *Sorghum bicolor* (sorghum) (C), and *Oryza Sativa* (rice) (D): the top graph plots the prediction probabilities of the PF-NET predictions for each intersection, while the bottom bar graph plots the counts of each intersection and the horizontal bar graph on the bottom left shows the total predicted phosphatase proteins by each method.

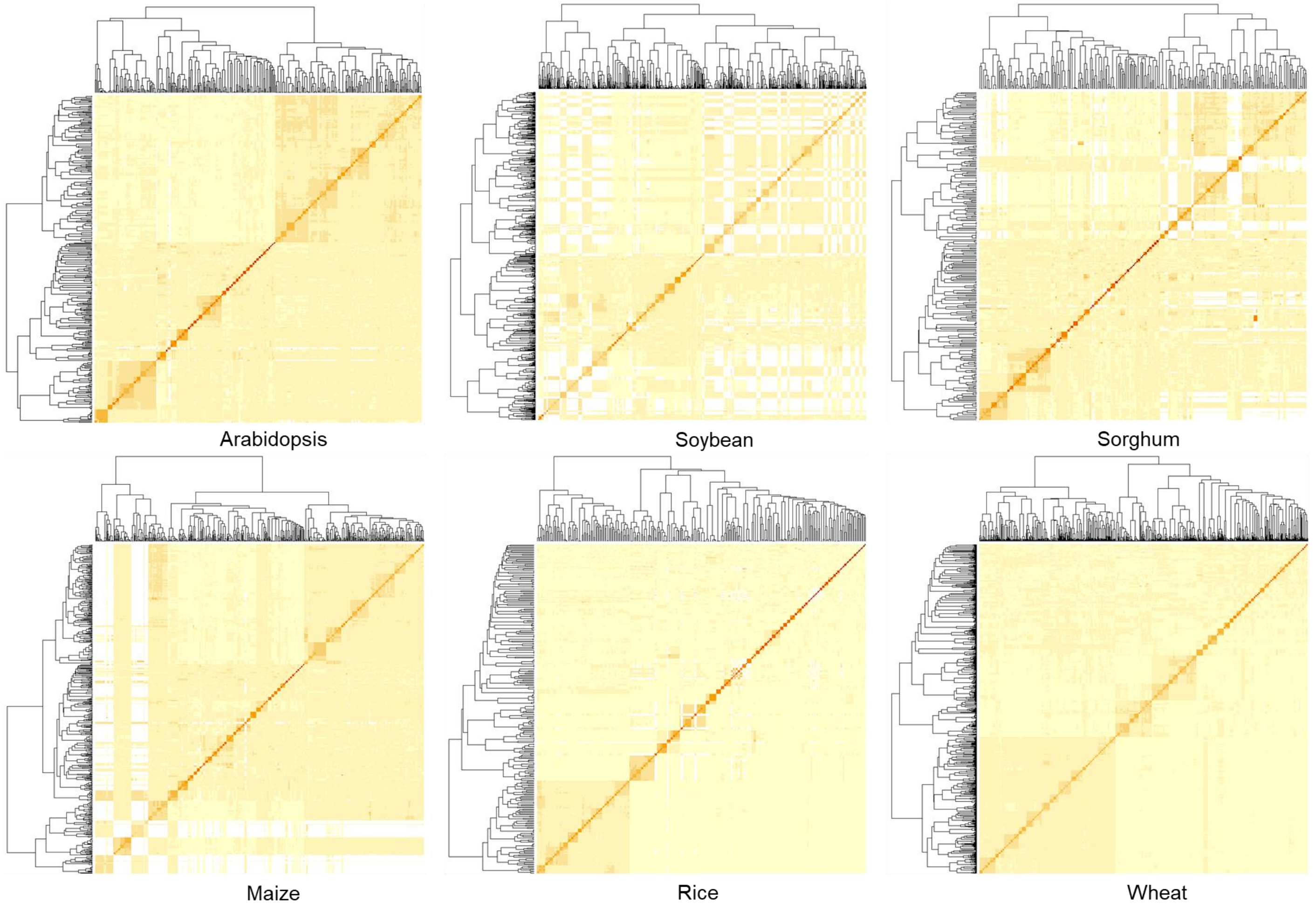

Supplementary Fig. S7 - Sequence percent identity matrices for phosphatases from Arabidopsis, soybean, sorghum, maize, rice, and wheat. The axes contain all the predicted phosphatases clustered according to hierarchical clustering. Color scale ranges from white (minimum value) to orange (maximum value).

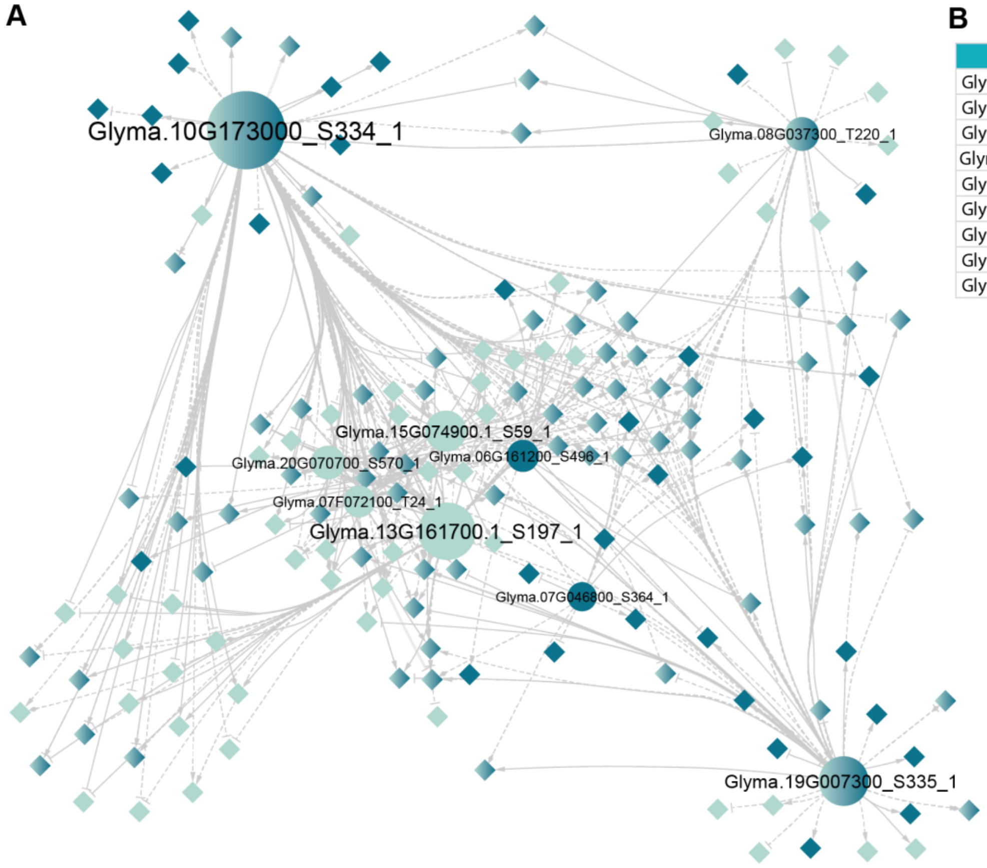

B

Protein	Best BLAST Ath	Centrality	Rewiring
Glyma.10G173000	TOI5	1.265	62.5
Glyma.19G007300	TOT3	1.029	34.5
Glyma.08G037300	CDKF;1	0.259	15.5
Glyma.13G161700	CRCK3	0.167	44
Glyma.06G161200	CPK4	0.057	13
Glyma.15G074900	AT5G58950	0.007	24
Glyma.20G070700	CPK1	0.002	16
Glyma.07G046800	SRF6	0	9
Glyma.07G072100	BSK3	0	13.5

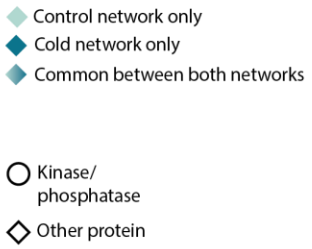

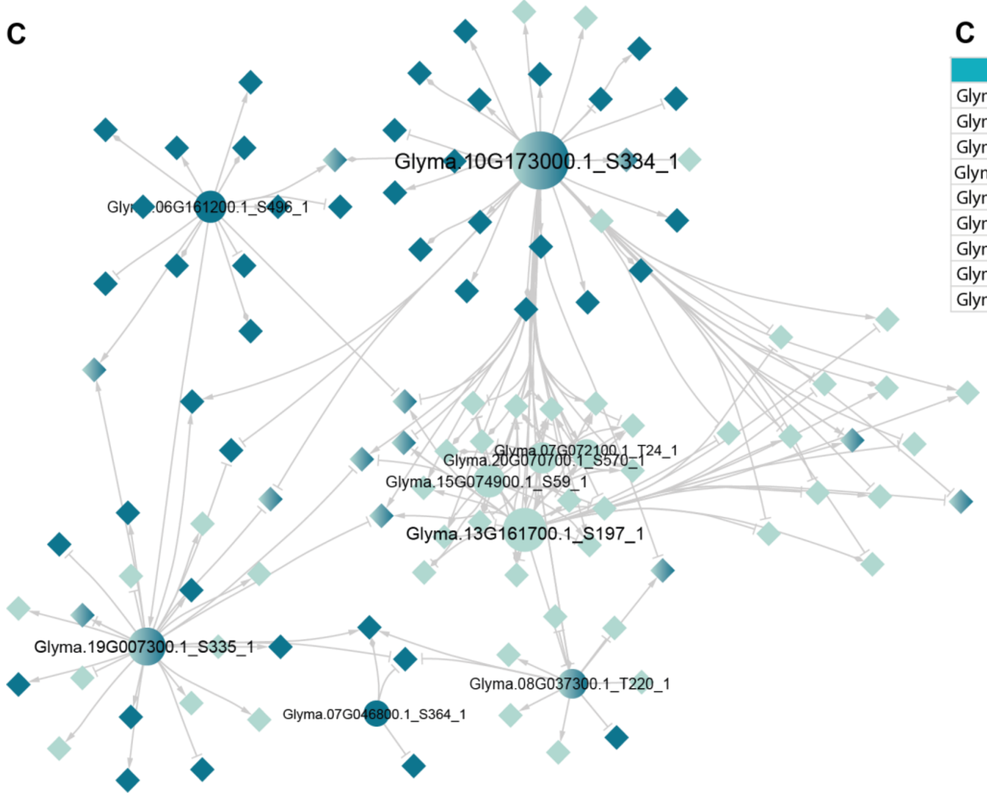

C

Protein	Best BLAST Ath	Centrality	Rewiring
Glyma.10G173000	TOI5	0.599	35
Glyma.19G007300	TOT3	0.201	14
Glyma.08G037300	CDKF;1	0.218	6.5
Glyma.13G161700	CRCK3	0.206	21.5
Glyma.06G161200	CPK4	0	7.5
Glyma.15G074900	AT5G58950	0.004	9
Glyma.20G070700	CPK1	0.006	8
Glyma.07G046800	SRF6	0	1.5
Glyma.07G072100	BSK3	0	5.5

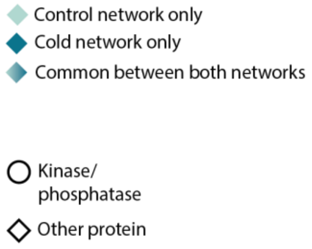

Supplementary Fig. S8 - Signaling pathways upon cold stress in soybean Altona. (A,C) Causal relations for cold and controlled conditions were predicted with a dynamic Bayesian network approach between differentially phosphorylated kinases/phosphatases and downstream phosphosites including both the simultaneous and antecedent (A) or only the antecedent (C) phosphorylation change of the kinases/phosphatases when compared with the phosphorylation change of their potential targets. Gray, dark blue, and merged gray/dark blue nodes represent phosphosites present in the control, cold, or both networks, respectively. Round and triangle nodes represent kinases/phosphatases and other phosphosites, respectively. (B, D) The betweenness centrality and rewiring value calculated in Cytoscape for each of the upstream nodes from the networks in panel A (B) and panel C (D). In the reduced network from panel C, TOI5 still showed the highest centrality and rewiring upon cold. TOT3 became the fourth most central node instead of the second most central, but still showed the third highest rewiring upon cold.

Supplementary Table S1 - Best performing hyperparameters.

Hyperparameter	Value
**Optimizer**	Nadam
**Learning rate**	$1E^{-7}$
**Dropout probabilities**	0.3, 0.2, 0.5
**Filters**	320
**Batch size**	100
**Activation function**	softmax
**Epochs**	60 (12 per batch)
**Alpha (focal loss)**	0.45
**Gamma (focal loss)**	2

Supplementary Table S2 - Comparison between NetPhorce and Perseus at various intermediate steps of the data analysis of the soybean cold phosphoproteomics.

	Perseus	NetPhorce
**Potential contaminant and Reverse (phosphopeptides)**	8081	8081
**Valid values filtering (phosphopeptides)**	361	361
**Valid values filtering (proteins)**	320	320
**Statistics (phosphopeptides)**	253 (70.1%)	310 (85.9%)
**Absence/Presence**	/	11
