# Supplementary material for: Functional annotation of proteins for signaling network inference in non-model species
Source: Nat Commun. 2023 Aug 3;14:4654. doi: 10.1038/s41467-023-40365-z (PMC10400656; doi:10.1038/s41467-023-40365-z)
Supplement: Supplementary file 3 — Description of Additional Supplementary Files [file 41467_2023_40365_MOESM3_ESM.pdf]

Description of Additional Supplementary Files

Supplementary Data 1. Identified phosphatases in soybean according to PF-NET, HMMER, and Arabidopsis orthologs (soybean genome reference assembly 2 version 1).

Supplementary Data 2. Overview of the 996 protein families with their respective precision, recall, f1-score, AUPR, and number of sequences in the test dataset.

Supplementary Data 3. Identified kinases and phosphatases in yeast according to PF-NET, HMMER, and the ground truth, here represented by a manually curated list of biochemical experimentally validated kinases and phosphatases.

Supplementary Data 4. PF-NET predictions for the entire Arabidopsis thaliana proteome.

Supplementary Data 5. PF-NET predictions for the entire soybean proteome (reference: assembly 2 annotation 1).

Supplementary Data 6. Gene information collected from soybase of the newly predicted soybean kinases (sheet 1) and phosphatases (sheet 2) by PF-NET.

Supplementary Data 7. Output table upon analysis with NetPhorce. Label-free phosphoproteome data from soybean var. Altona was collected every 6 minutes for 1 hours upon cold and control treatment. This data was analyzed using NetPhorce, which performs data quality controls and statistical analysis (see methods for more information).

Supplementary Data 8. Network regulations upon cold and controled conditions in soybean. Causal regulations between phosphorylated proteins were identified using NetPhorce. The pipeline leverages a time course to infer causal regulation based on dynamics Bayesion principles (see methods for more information).

Supplementary Data 9. PF-NET predictions for the entire maize, sorghum, wheat, and rice proteome.

Supplementary Data 10. Phylogenetic analysis of Arabidopsis thaliana, Glycine max (soybean), Triticum aestivum (wheat), Zea mays (maize), Sorghum bicolor (sorghum), and Oryza sativa (rice). The predicted phosphatases from PF-NET and HMMER and their orthogroup are listed.
