# Supplementary material for: Functional annotation of proteins for signaling network inference in non-model species
Source: Nat Commun. 2023 Aug 3;14:4654. doi: 10.1038/s41467-023-40365-z (PMC10400656; doi:10.1038/s41467-023-40365-z)
Supplement: Supplementary file 14 — Reporting Summary [file 41467_2023_40365_MOESM14_ESM.pdf]

nature portfolio

Corresponding author(s): Rosangela Sozzani, Lisa Van den Broeck

Last updated by author(s): Nov 8, 2022

Reporting Summary

Nature Portfolio wishes to improve the reproducibility of the work that we publish. This form provides structure for consistency and transparency in reporting. For further information on Nature Portfolio policies, see our Editorial Policies and the Editorial Policy Checklist.

Statistics

For all statistical analyses, confirm that the following items are present in the figure legend, table legend, main text, or Methods section.

n/a	Confirmed	
☐	☒	The exact sample size (*n*) for each experimental group/condition, given as a discrete number and unit of measurement
☐	☒	A statement on whether measurements were taken from distinct samples or whether the same sample was measured repeatedly
☐	☒	The statistical test(s) used AND whether they are one- or two-sided<br>*Only common tests should be described solely by name; describe more complex techniques in the Methods section.*
☒	☐	A description of all covariates tested
☐	☒	A description of any assumptions or corrections, such as tests of normality and adjustment for multiple comparisons
☐	☒	A full description of the statistical parameters including central tendency (e.g. means) or other basic estimates (e.g. regression coefficient) AND variation (e.g. standard deviation) or associated estimates of uncertainty (e.g. confidence intervals)
☐	☒	For null hypothesis testing, the test statistic (e.g. *F*, *t*, *r*) with confidence intervals, effect sizes, degrees of freedom and *P* value noted<br>*Give P values as exact values whenever suitable.*
☐	☒	For Bayesian analysis, information on the choice of priors and Markov chain Monte Carlo settings
☒	☐	For hierarchical and complex designs, identification of the appropriate level for tests and full reporting of outcomes
☒	☐	Estimates of effect sizes (e.g. Cohen's *d*, Pearson's *r*), indicating how they were calculated

Our web collection on statistics for biologists contains articles on many of the points above.

Software and code

Policy information about availability of computer code

Data collection	Orthologs were identified with PLAZA 4.5 through the Plaza Integrative Method (https://bioinformatics.psb.ugent.be/plaza/) and orthogroups with Orthofinder (version 2.5.4). All sequences found in Pfam's underlying sequence database of each Pfam family were extracted using MySQL workbench. The script used is available at https://github.com/LisaVdB/PF-NET. Kinases and phosphatases were identified with HMMER (https://www.ebi.ac.uk/Tools/hmmer/search/hmmsearch) (HmmerWeb version 2.41.2).
Data analysis	MS/MS spectra files were searched against the Soybean database with Maxquant software version 1.6.10.43. Source code of the neural network PF-NET is available at https://github.com/LisaVdB/PF-NET. A webtool was also built to easily make predictions with PF-NET (https://sozzanilab.shinyapps.io/PF-NET_Shiny/). Source code and instructions for the phosphoproteomics analysis and Bayesian inference are available at https://ksong4.github.io/NetPhorce/. All required packages and their versions are installed through the NetPhorce R package. Figures were made in R (version 4.2.2) using ggplot2 (version 3.4.1), or networkD3 (version 0.4) and htmlwidgets (version 1.6.1) for the Sankey diagram. Statistical tests are listed in the methods under "Analysis and network inference with NetPhorce".

For manuscripts utilizing custom algorithms or software that are central to the research but not yet described in published literature, software must be made available to editors and reviewers. We strongly encourage code deposition in a community repository (e.g. GitHub). See the Nature Portfolio guidelines for submitting code & software for further information.

Data

Policy information about availability of data

All manuscripts must include a data availability statement. This statement should provide the following information, where applicable:

- Accession codes, unique identifiers, or web links for publicly available datasets
- A description of any restrictions on data availability
- For clinical datasets or third party data, please ensure that the statement adheres to our policy

The mass spectrometry proteomics data, the MaxQuant settings, MaxQuant outputs and resulting merged file generated in this study have been deposited in the ProteomeXchange Consortium via the PRIDE database under accession code PXD037601. The prediction data, PF-NET's performance, network inference output, and orthogroup data generated in this study are provided in the Supplementary Information. Sequences from Pfam's underlying sequence database of each Pfam family used in this study were extracted from http://ftp.ebi.ac.uk/pub/databases/Pfam/. The yeast, A. thaliana, soybean, wheat, sorghum, rice, and maize proteome used in this study were downloaded at the Saccharomyces Genome Database (https://www.yeastgenome.org/), TAIR (https://arabidopsis.org/), Soybase (https://www.soybase.org/), EnsemblPlants (https://plants.ensembl.org/Triticum_aestivum/Info/Index), EnsemblPlants (https://plants.ensembl.org/Sorghum_bicolor/Info/Index), rice genome annotation project (http://rice.uga.edu/), and Maize Genome Database (https://www.maizegdb.org/).

Human research participants

Policy information about studies involving human research participants and Sex and Gender in Research.

Reporting on sex and gender	NA
Population characteristics	NA
Recruitment	NA
Ethics oversight	NA

Note that full information on the approval of the study protocol must also be provided in the manuscript.

Field-specific reporting

Please select the one below that is the best fit for your research. If you are not sure, read the appropriate sections before making your selection.

☒ Life sciences ☐ Behavioural & social sciences ☐ Ecological, evolutionary & environmental sciences

For a reference copy of the document with all sections, see nature.com/documents/nr-reporting-summary-flat.pdf

Life sciences study design

All studies must disclose on these points even when the disclosure is negative.

Sample size	Three distinct plants were pooled for phosphoproteomics, which was repeated four times for four biological replicates.
Data exclusions	Phosphosites with insufficient data points due to detection limits across the time course were excluded from downstream analyses.
Replication	Three distinct plants were pooled for phosphoproteomics, which was repeated four times for four biological replicates.
Randomization	Because the treatment is applied by transferring plants to different temperature chambers, randomization between treatments was not possible.
Blinding	Due to the setup of the experiment using different temperature chambers, blinding was not possible.

Reporting for specific materials, systems and methods

We require information from authors about some types of materials, experimental systems and methods used in many studies. Here, indicate whether each material, system or method listed is relevant to your study. If you are not sure if a list item applies to your research, read the appropriate section before selecting a response.

Materials & experimental systems

n/a	Involved in the study
☒	☐ Antibodies
☒	☐ Eukaryotic cell lines
☒	☐ Palaeontology and archaeology
☒	☐ Animals and other organisms
☒	☐ Clinical data
☒	☐ Dual use research of concern

Methods

n/a	Involved in the study
☒	☐ ChIP-seq
☒	☐ Flow cytometry
☒	☐ MRI-based neuroimaging
